# Snare technique is useful for leadless pacemaker implantation in a patient with severe right atrial dilatation

**DOI:** 10.1002/joa3.70075

**Published:** 2025-04-23

**Authors:** Kosuke Hirose, Tomoki Fukui, Miwa Miyoshi, Nobuyuki Ogasawara

**Affiliations:** ^1^ Department of Cardiology Japan Community Healthcare Organization (JCHO) Osaka Hospital Osaka Japan

**Keywords:** atrial fibrillation, bradycardia, heart failure, pacemaker

## Abstract

Leadless pacemaker implantation in a patient with severe right atrium dilation was unsuccessful using the conventional approach. The delivery system failed to gain sufficient backup force from the atrial wall and moved upward within the dilated atrium. To overcome this, the snare technique was employed. By securing the slightly distal portion of the top of the shaft curve, the pushing force was effectively transmitted to the tip of the system, creating a stable gooseneck shape for successful implantation.

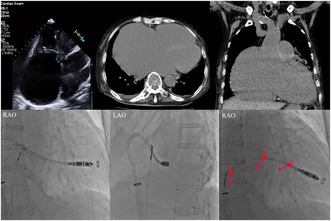

## 1 SPOTLIGHT

**FIGURE 1:**
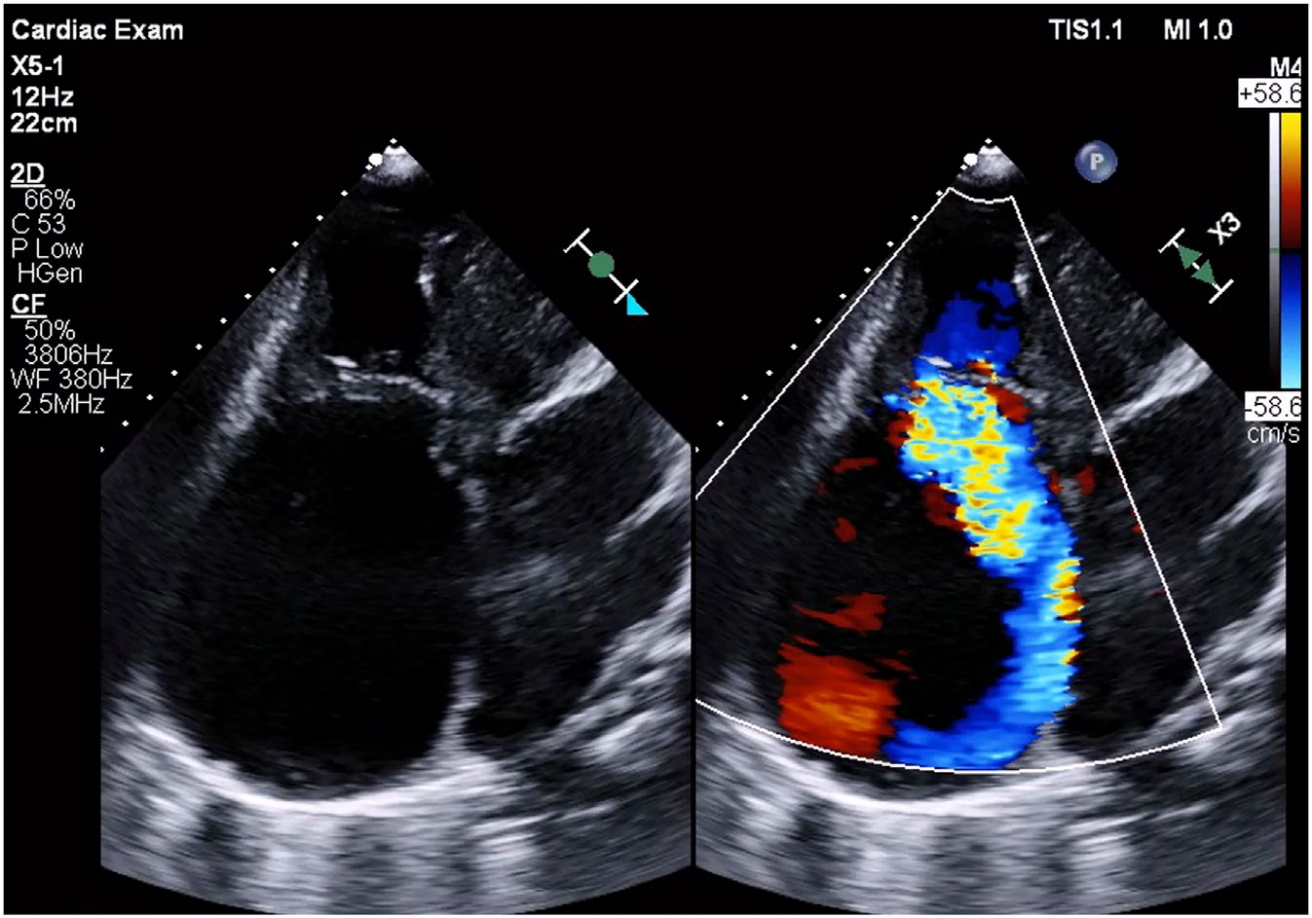
Echocardiography showing severe right atrial dilatation and severe tricuspid regurgitation.

**FIGURE 2:**
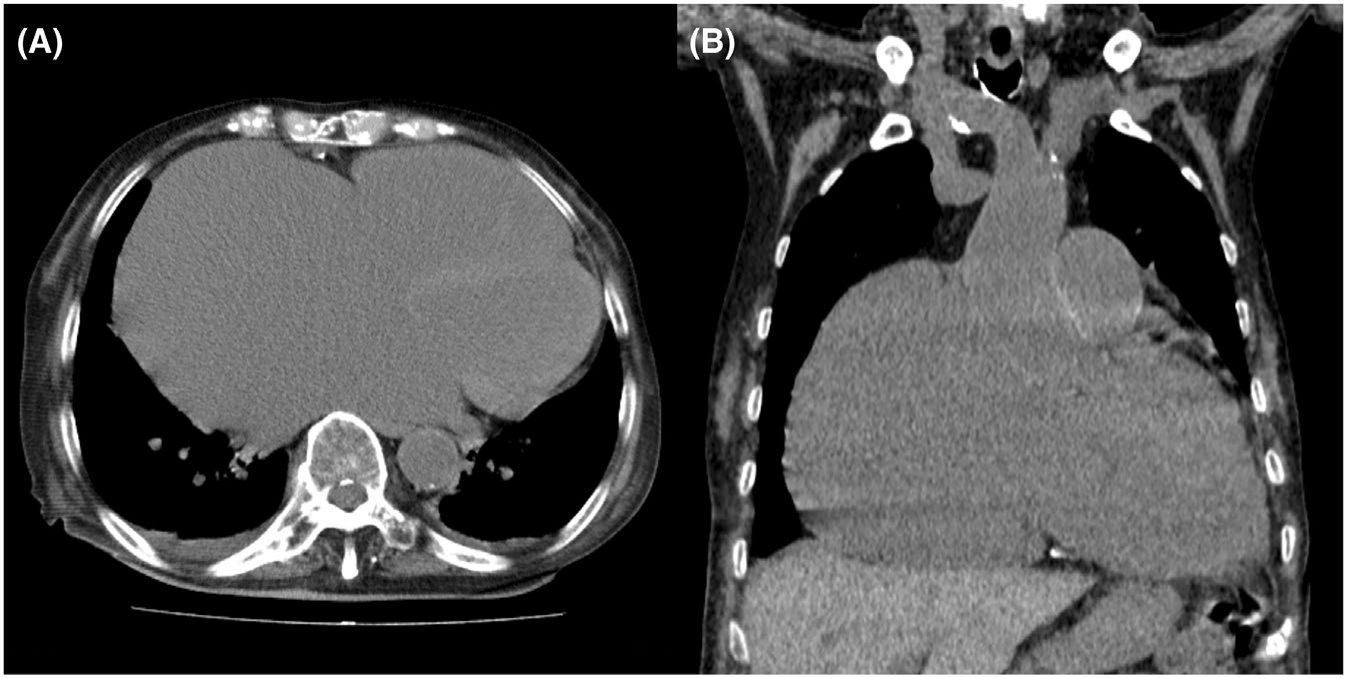
Computed tomography images indicating severe enlargement of the right atrium. (A) Axial view. (B) Coronal view.

**FIGURE 3:**
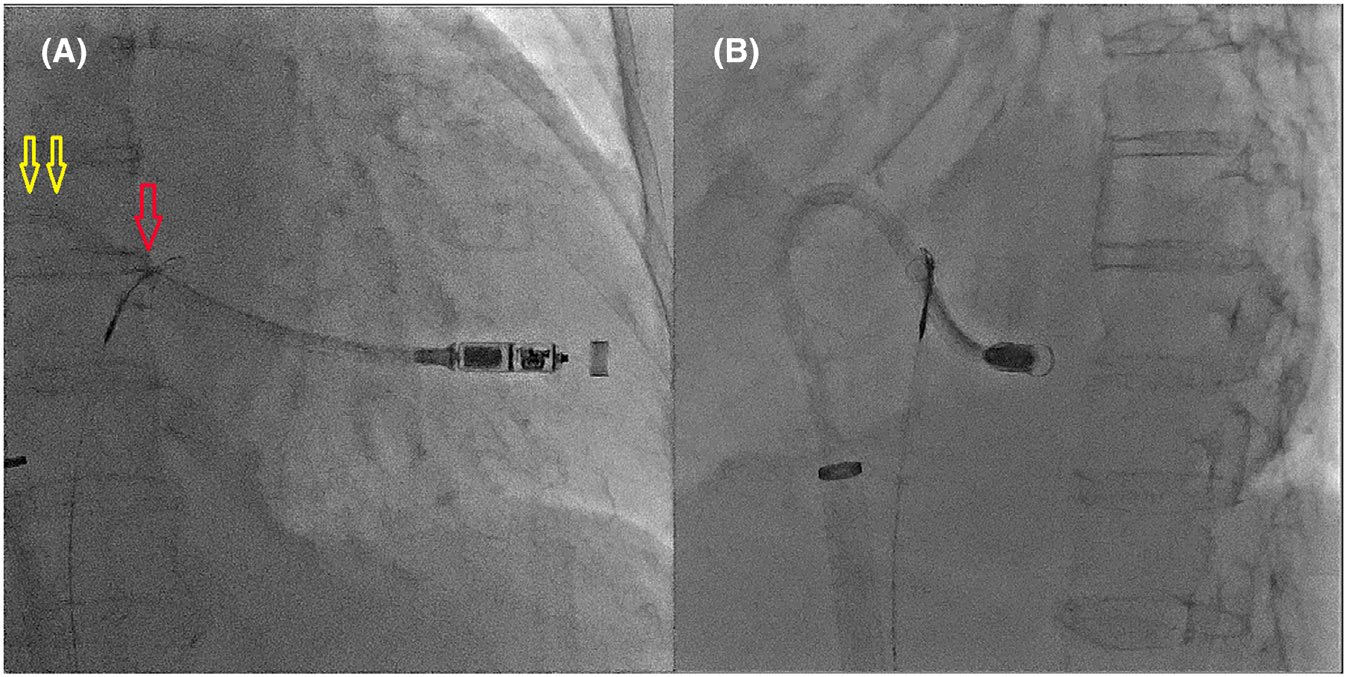
Snare‐assisted technique demonstrating transmission of the pushing force to the system tip, creating a gooseneck shape. (A) RAO view. The red arrow points to the snare. The yellow arrows point to the top of the shaft's curve. (B) LAO view.

An 83‐year‐old woman with chronic heart failure with preserved ejection fraction, chronic atrial fibrillation, severe tricuspid regurgitation, and dementia was admitted to our hospital because of worsening heart failure. Electrocardiography revealed a junctional rhythm of 44 beats/min. Chest radiography revealed cardiomegaly and a new right pleural effusion. Echocardiography indicated severe right atrial dilatation (right atrial volume index: 414 mL/m^2^), mitral regurgitation, and tricuspid regurgitation with pulmonary hypertension (Figure 1). Left ventricular wall motion remained normal, with a left ventricular ejection fraction of 66%, measured using the Teichholz formula. Computed tomography confirmed severe right atrium enlargement (Figure 2). A leadless pacemaker (Micra; Medtronic, Inc., Minneapolis, MN, USA) was selected over a transvenous pacemaker because of the patient's dementia, which hindered postoperative wound management. Despite successful delivery into the right ventricle, the pacemaker system failed to gain adequate backup force from the atrial wall because of severe right atrial dilatation. Pressing the delivery system on the right ventricular septum caused the catheter to move upward into the right atrium without transmitting force to the catheter tip. Even when the pacemaker was positioned, it dislodged easily. Pacemaker implantation using the conventional technique was unsuccessful. To transmit the pushing force to the catheter tip, a downward force was required. A snare (12–20 mm Atrieve Vascular Snare Kit; Argon Medical Devices Inc., Athens, TX, USA) was inserted from the opposite femoral vein to address this issue. The snare captured the shaft of the system, allowing the pulling force to be transmitted effectively to the tip, thereby creating a gooseneck shape (Figure 3, Videos S1 and S2). This modification enabled successful pacemaker implantation without complications.

Herein, we report a case in which a leadless pacemaker was successfully implanted using a snare technique in a patient with severe right atrial dilatation. Although leadless pacemaker implantation has a high success rate,^1^ it is challenging in patients with marked skeletal deformities, complex cardiac anatomy, or limited venous access. Severe right atrial dilation is an anatomical abnormality that complicates implantation. In a previous report, leadless pacemaker implantation was unsuccessful, and the device was replaced with a conventional transvenous pacemaker.^2^ For patients with chronic atrial fibrillation and significant right atrial enlargement, the snare technique has proven effective, although reports of its use in leadless pacemaker implantation are rare. Herein, we provide insights into this technique.

Several methods for crossing the delivery system with a snare have been described. Alyesh et al. positioned the snare on the delivery catheter shaft proximal to the pacing capsule while the catheter was outside the body.^3^ This method is straightforward; however, using another device through the same sheath may result in backflow of blood and inflow of air. Alternatively, the snare‐kissing catheter technique involved crossing a single‐loop snare with the delivery system in the inferior vena cava.^4^ However, this approach is challenging because of the thickness of the delivery system tip. To address this, a tapered dilator was used to facilitate snare capture of the sheath, enabling the subsequent positioning of the snare on the shaft. It should be noted that with our method, compared to conventional leadless pacemaker implantation, there is a risk of complications such as vascular injury, thrombosis, arterial puncture, and bleeding associated with the additional puncture of the femoral vein. Furthermore, because this is a procedure that cannot be performed by a single person, increased collaboration with the assistant operator who holds the snare is necessary.

**FIGURE 4:**
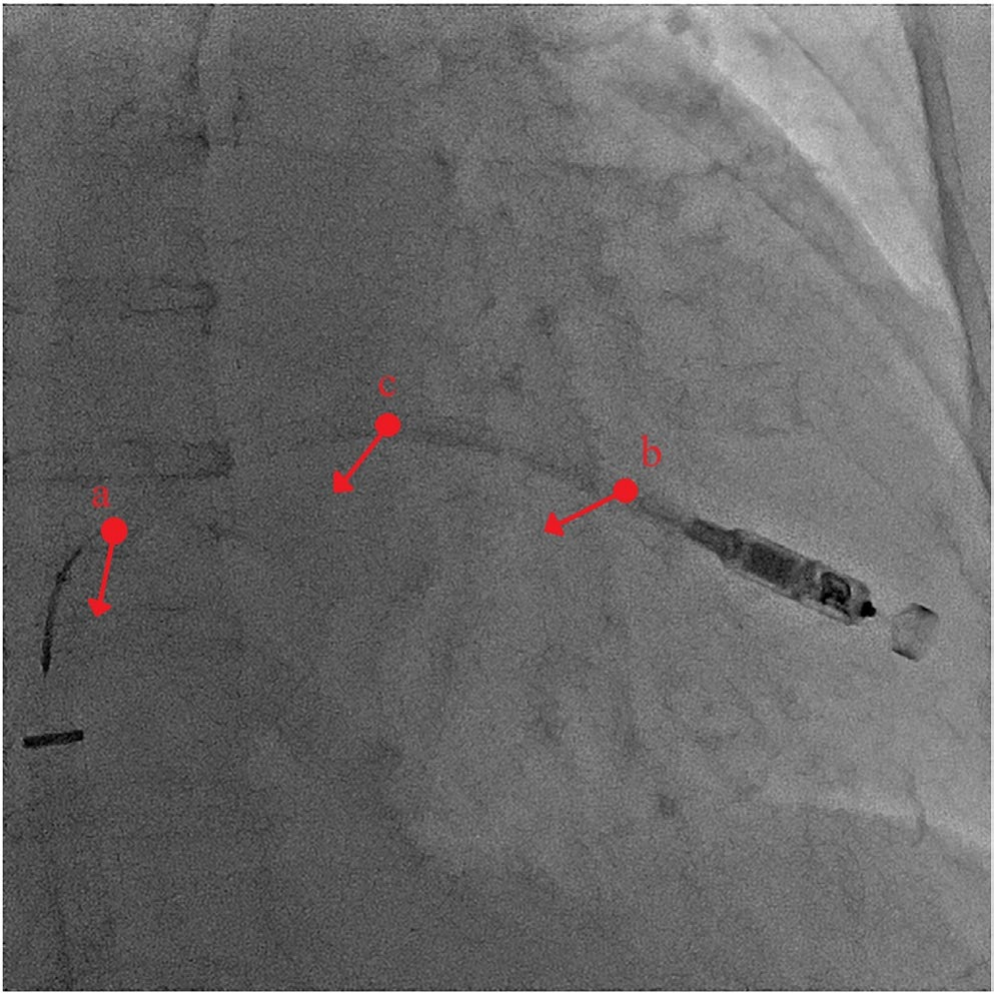
Relationship between the gripping location and the transmission of pulling force: (a) proximal part of the shaft, (b) distal part of the shaft, and (c) slightly distal part of the top of the shaft's curve. The force was successfully transmitted to the tip at point (c).

The location where the catheter is caught with the snare is critical. Capturing the proximal shaft prevents effective pushing (Figure 4, a). Capturing the distal part of the shaft applies a horizontal pulling force (Figure 4, b), which does not transmit sufficient force to the tip, causing the system to move upward. Capturing the slightly distal part of the shaft curve transmits the pushing force to the tip, creating a gooseneck shape and ensuring successful deployment (Figure 4, c, Videos S1 and S2).

In conclusion, the snare technique provides a viable solution to achieve sufficient tip force during leadless pacemaker implantation in patients with severe right atrial dilatation.

## FUNDING INFORMATION

We confirm that we have not received any funding for the above study.

## CONFLICT OF INTEREST STATEMENT

The authors declare that there is no conflict of interest.

## PATIENT CONSENT STATEMENT

The patient has provided consent for publication.

## Supporting information


**Video S1.** This is a video showing the RAO view of an actual pacemaker implantation.

**Video S2.** This is a video showing the LAO view of an actual pacemaker implantation.

## Data Availability

Most data associated with this study are provided in the text and figures. Additional data are available upon request.
